# Health care workers’ qualitative descriptions of ethically challenging situations evoking moral distress during Covid-19

**DOI:** 10.1177/09697330241257567

**Published:** 2024-06-10

**Authors:** Kristin Alve Glad, Hilde Wøien, Synne Øien Stensland, Solveig Klebo Reitan, John Anker Henrik Zwart, Dan Atar, Grete Dyb, Kristina Bondjers

**Affiliations:** 25566 Norwegian Centre for Violence and Traumatic Stress Studies; 155272 Oslo University Hospital; 6305 University of Oslo; 25566 Norwegian Centre for Violence and Traumatic Stress Studies; 155272 Oslo University Hospital; 8018 NTNU; St Olav University Hospital; 155272 Oslo University Hospital; 6305 University of Oslo; 6305 University of Oslo; 25566 Norwegian Centre for Violence and Traumatic Stress Studies; 6305 University of Oslo; 25566 Norwegian Centre for Violence and Traumatic Stress Studies

**Keywords:** COVID-19, ethically challenging situations, healthcare workers, moral distress, pandemic

## Abstract

**Background:**

The high public demand for healthcare services during the COVID-19 pandemic and strict infection control measures, coupled with threat of severe illness and death, and limited resources, led to many healthcare workers (HCWs) experiencing ethically challenging situations (ECSs).

**Objective:**

To systematically explore first-hand accounts of ECS-evoking moral distress among HCWs during this public health emergency.

**Research design:**

This was an open cohort study. All participants were asked whether they had been in ECS-evoking moral distress during the pandemic. Those who had were asked to describe these situations. Answers were systematically analyzed according to three levels of root causes for ECSs, using thematic analysis.

**Participants and research context:**

In January 2022, 977 HCWs from four Norwegian university hospitals participated.

**Ethical considerations:**

The study received ethical approval from the Norwegian Ethical Review Authority (No. 130944).

**Results:**

In total, 508 participants (52%) reported that they had experienced ECS-evoking moral distress during the pandemic, whereof 323 provided a qualitative description. We found that while a few reported ECSs caused at the patient level, and some described situations at the unit/team level, the vast majority reported situations caused at the system level, predominantly related to resource scarcity, particularly poor staffing.

**Conclusion:**

Our findings strongly indicate that efforts to mitigate moral distress among HCWs should be targeted at the system level. More specifically, the study findings highlight resource limitations, particularly poor staffing, as a major cause of moral distress during the pandemic.

## Introduction

The high public demand for healthcare services and strict infection control measures during the Coronavirus Disease-2019 (COVID-19) pandemic, coupled with threat of severe illness and death in patients and personnel, as well as limited resources, led to many healthcare workers (HCWs) experiencing ethically challenging situations (ECSs).^1^ Stress and discomfort related to ECSs have been referred to as “moral distress.”^2^ Moral distress arises when an individual recognizes the right course of action, but institutional constraints impede his/her ability to follow through with that action.^3^ More specifically, according to Epstein and Hamric et al., “moral distress is the result of a perceived violation of one’s core values and duties, concurrent with a feeling of being constrained from taking ethically appropriate action” (p.2).^4^ Organizational and individual preparation before exposure to ECSs, as well as formal and informal support structures in the aftermath, has been suggested to potentially alleviate moral distress.^5^ However, to enable implementation of successful measures to mitigate moral distress, it is important to identify situations that evoke it. In the present study, we systematically explore situations described by HCWs themselves as ethically challenging and evoking moral distress during the COVID-19 pandemic.

## Background

It has been suggested that most ECSs can be attributed to root causes at three different levels.^6^ Causes at the s*ystem level* include consistently poor staffing and lack of adequate resources, such as supplies or bed capacity.^7^ At the *unit/team level*, ECSs may arise as a result of poor communication or collaboration between team members, impacting patient care (e.g., witnessing false hope and being bullied by colleagues). Causes at the *patient level* involve the patients or their families (e.g., relatives demanding specific treatments that cause unnecessary suffering or that contradict current scientific recommendations).^7^

High prevalence of moral distress has been reported during the COVID-19 pandemic. For example, Norman et al. found that, in a sample of more than 2500 frontline healthcare nurses caring for patients with COVID-19, a majority (52.7–87.8%) had experienced moral distress.^8^ Situations identified as causing moral distress among HCWs during the pandemic include being unable to provide adequate emotional support for patients and their families,^9^ resource scarcity,^10,11^ personal protective equipment (PPE) limiting their ability to provide adequate care to patients, fear of letting co-workers down by becoming infected,^11^ worry about infecting patients or colleagues with COVID-19,^8^ transmission risk to family members, caring for patients without family members present, and caring for dying patients without family members or clergy present.^8,11^ However, as stressed by Silverman et al., potential causes of moral distress during the COVID-19 pandemic have not been sufficiently explored, and a qualitative approach may offer important insights.^12^ Using focus group discussions and in-depth interviews with nurses caring for patients with COVID-19, they identified several specific causes of moral distress related to the pandemic, including fear of exposure to the virus leading to suboptimal care, and policies to reduce viral transmission that prevented nurses from fulfilling their caring role.^12^

In sum, whereas many researchers have explored moral distress among HCWs, few have explored what type of situations HCWs themselves describe as ethically challenging during a public health emergency. To enable implementation of successful measures to alleviate moral distress among HCWs, it is important to identify the situations that evoke it. In the present study, we aimed to systematically explore first-hand accounts of the causes of ECSs leading to moral distress in a large sample of frontline HCWs during the COVID-19 pandemic.

## Method

The current study derives data from a comprehensive longitudinal open-cohort study exploring the impact of the COVID-19 pandemic on work environment, professional quality of life, and the health of HCWs at four large university hospitals in Norway throughout the four waves of the COVID-19 pandemic. This paper uses data from the fourth data collection (T4), in January/February 2022.

### Participants and procedure

Eligible participants were employees at four large university hospitals in Norway. Invitations to participate in a web-based survey were sent out via the hospitals’ typical channels for communicating with their staff (e.g., e-mail, SMS, online bulletin boards, and posters). In total, 977 HCWs (75% female) participated at the fourth assessment point. Of these, 45% were nurses, 19% physicians, and 36% were other hospital personnel (e.g., physiotherapists, assistant nurses, ambulance personnel, and psychologists). All participants were given the following definition of moral distress: “*Distress or worry you feel when you know what the ethically right thing to do is*, *but various obstacles (*e.g., *lack of personnel resources*, *lack of equipment*, *procedures*, *pressure from others) prevent you from doing it*” and asked if they had experienced situations evoking this type of response during the pandemic. Participants who answered yes, received a follow-up question: “*In what situations have you experienced this*, *and how did you handle it*?” and were prompted to provide a written response. Due to a low response rate on the second part of this question, results on participants’ coping responses are not included in the present paper.

### Ethical considerations

Informed consent was obtained from all subjects. Participants were informed that participation was voluntary and that they could withdraw their consent at any time, without any consequences. Further, they were informed that their response to the questionnaire was sent directly to a secure server and that the information was accessible only to researchers working within the project, all of whom have a duty of confidentiality. Participants were also informed that the participating hospitals would provide information to employees about access to help and support during the pandemic, and that if the study would uncover that extra measures were needed to improve working conditions, this would be shared with employee representatives, safety delegates, and management at the hospital, while ensuring anonymity for the participants. Finally, they were provided with the contact information of the study’s primary investigator and informed that if they had any questions, or wanted to withdraw from the study, they were welcome to contact her. The study received ethical approval from the Norwegian Ethical Review Authority (ref no. 130944). In order to secure anonymity, all potentially identifiable personal information in the participants’ descriptions of ECSs included in the present paper has been removed or modified.

### Analyses

The HCW’s written descriptions of ECSs during the COVID-19 pandemic were analyzed following the principles of thematic analysis.^13^ First, to become familiar with the data and generate initial ideas, the first (KAG) and last (KB) authors carefully read and wrote reflective notes regarding the causes of situations described. KAG and KB subsequently discussed the content of the responses and possible codes. As a next step, KAG systematically organized all the data into units, or codes, based on the nature of the situations described. The participants’ descriptions were divided into 390 unique codes using Microsoft Excel 2016. Each discourse unit included a description of an ECS. This is in line with Boyatzis’ definition of a code: “the most basic segment, or element, of the raw data or information that can be assessed in a meaningful way regarding the phenomenon” (p. 63).^14^ Then, the codes were identified both deductively, based on the framework for ECSs presented by Hamric and Epstein,^6^ and inductively, from the data. The analysis was abductively driven; we moved back and forth between inductive and deductive approaches during different stages in the analysis. Suggested themes and subthemes were subsequently presented to, and discussed with, KB and the second author (HW). When a proposed categorization was agreed upon, the remaining authors gave their input. The analysis was finalized when all the authors agreed upon the structure and theme names, and illustrative quotes had been selected.

In the initial reading of the qualitative material of the 344 participants who provided a written description, KAG and KB found that 21 individuals had not directly answered the research question or that their answers were difficult to interpret and code. Thus, the qualitative results coded for this study are based on reports from 323 HCWs. All quotes presented here have been translated from Norwegian to English by a professional translation agency (Semantix).

## Results

Of the 977 HCWs who participated in the present study, 508 (52%) reported that they had experienced ECSs during the pandemic. Of these, 323 provided a written description of their experiences. Through our coding of these responses, we identified eight themes which aligned with the three levels of root causes for ECSs (i.e., system, unit/team, and patient) described by Hamric and Epstein (2017). In the following, findings are organized according to these three broad levels, with example quotes which reflect the identified themes and subthemes (for an overview, see Table 1).

**Table 1.** Levels, themes, and subthemes in the causes of the ethically challenging situations experienced by healthcare workers (*n* = 323) during COVID-19.

Root cause	Theme	Subtheme	N^a^ (%)
System level			266 (82.4)
	Inadequate resources		206 (63.8)
		Poor staffing	134 (41.5)
		Insufficient bed capacity	51 (15.8)
		Lack of equipment/rooms	39 (12.1)
	Challenging COVID-19 regulations		69 (21.4)
		Isolation	23 (7.1)
		Family visitation restrictions	21 (6.5)
		Digital follow-up	13 (4.0)
		PPE usage	7 (2.2)
		In-house cohorts	4 (1.2)
	General situation		9 (2.8)
Unit/team level			61 (18.9**)**
	COVID-19-specific issues		26 (8.1)
		Unclear information and routines	11 (3.4)
		Fear of infection	9 (2.8)
		Reallocated staff’s competence	6 (1.9)
	Choice of treatment level		23 (7.1)
	Mismanagement		12 (3.7)
		Insufficient qualification	6 (1.9)
		Routines not followed	5 (1.5)
		Lack of support	2 (0.1)
Patient level			9 (2.8)
	Patient autonomy		5 (1.5)
	Unvaccinated COVID-19 patients		4 (1.2)

^a^Some participants described more than one theme or subtheme. Thus, the sum totals for each root cause, theme, and subtheme exceed the number of participants in the study who reported having experienced an ethically challenging situation.

### System level

At the system level, we identified three main causes of ECS: (1) inadequate resources, (2) challenging COVID-19 regulations, and (3) the general situation in the hospital.

**Inadequate resources** included ECSs related to poor staffing, insufficient bed capacity, and lack of equipment/rooms.

#### Poor staffing

was the most prominent cause of ECSs described by the participants. In particular, the participants described being overloaded and not having enough time for their patients. For example, they said that they had not been able to meet their patients’ most basic needs, as described by one nurse:

Going home, thinking about everything I should have done and feeling guilty for not being able to do everything the way I would have wanted. Having to make patients wait to go to the bathroom, for example, because there isn’t enough time or aren’t enough people at work. It’s undignified for the patients. Feeling as though you’re letting yourself and your patients down.

The participants described how the quality of care suffered substantially because there were too few people at work. As one participant noted:

Too little time to provide adequate healthcare to patients, not enough time to “see” them, no opportunity to make a comprehensive health assessment or address the entire clinical picture before they leave the institution.

Poor staffing also affected HCW’s ability to meet their patients’ psychosocial needs, as described by one participant: “Having to leave patients who are lonely or scared because there aren’t enough people at work and we have other duties and patients that need to be seen to.” The staffing situation led to difficult decisions regarding prioritization of patients, and participants described not having enough time to follow-up with their patients’ next of kin, being left alone with patients even though they needed help, or having to do a colleague’s job.

Many participants also described situations in which they felt pressured to work. Some felt pressure to work overtime or to go to work even though they were ill. For example, one participant said, “Had a fever, a negative covid test, took paracetamol and went to work because of the high levels of absence.” Others described feeling that they should work overtime to protect their colleagues, one of whom stated, “Feeling as though there’s a moral pressure to accept shifts, as you don’t want your colleagues to work double shifts/have a difficult shift. Colleagues vomit/cry before starting work.” Several leaders also described ECSs related to having to order people to work. For example, one stated the following:

As a head of unit, I experienced significant psychological stress due to not having sufficient nursing staff available during certain periods as a result of high levels of absence and a lack of access to bank staff from HR. This meant we had to impose overtime orders. There were times when I felt as though I had to “kick someone who was already down” in order to keep things running.

A few leaders described ECSs related to having too few staff left in their unit because their staff had been reallocated to the COVID-19 unit. For example, one said, “Pressure to take in poor heart patients when we don’t have enough staff because we HAVE to release staff for the covid cohort.”

#### Insufficient bed capacity

Insufficient bed capacity also led to ECSs by affecting patient flow both inside and outside the hospital. Inside the hospital, this included situations wherein the intensive care unit was full, and patients had to be treated at general wards or be moved from the intensive care unit too soon. The participants described difficult decisions regarding who to prioritize for proper monitoring, one of whom stated the following:

When the intensive care unit is overcrowded and I’m on duty, it’s sometimes my responsibility to determine which patients to transfer to a lower care level (less specialized observation unit/ward) too soon or another hospital (resulting in less monitoring and increased risk during transport). Transfer will then take place despite the fact that it’s not in the best interests of the patient (risk and reduced expertise), but it’s necessary for us to admit the next patient and I then have to decide which patient to subject to such risks.

Outside the hospital, insufficient capacity resulted in patients not being admitted even though they needed to be, patients being moved between hospitals, and delayed treatments/long waiting lists. As one participant stated the following:

We’ve had to cancel scheduled operations for patients who have been waiting for several years, some of whom have experienced multiple cancellations. For some of these patients, we’re the only provider in Norway and their operations cannot be carried out anywhere else in the country. They feel frustrated and end up crying on the phone, even though they understand the situation, and all I can say is: “Apologies, we’ll schedule a new date for your operation as soon as we can.”

#### Lack of equipment and room facilitation

In the beginning of the pandemic, the lack of PPE led to poorer follow-up of patients with COVID-19 and hindered family visitation, as described by one participant: “Seriously ill covid patients were unable to see their relatives due to a lack of infection control equipment. This led to high levels of stress.” Another stated the following:

I’ve delayed attending to patients to make sure that the doctors have decided which blood samples they need to order to avoid any excessive use of infection control equipment. (…) Infection control is both time- and equipment-consuming.

Lack of PPE also put the staff at risk. For example, some leaders stated that they were forced to instruct their staff to reuse equipment: “At the start of the pandemic, stress levels were high due to a lack of access to infection control equipment and we were instructed to “order” employees to reuse P3 masks.” A few participants noted that the hospital rooms were inadequate and described ECSs in which patients had to be too close together, with the risk of infecting each other.

**Challenging COVID-19 regulations** included ECSs related to isolating patients with COVID-19 from non-COVID-19 patients, having to enforce family visitation restrictions, and difficulties related to digital follow-up, PPE usage, and in-house cohorts.

#### Isolation

Participants described that isolation of patients with COVID-19 led to them receiving poorer follow-up, as well as delayed assessments and treatment: “Patients have been left on their own for much longer than before for reasons of infection control: protective isolation.”

#### Family visitation restrictions

The participants described having to enforce visitation restrictions that were sometimes in conflict with the patients’ and their next of kins’ needs. This included patients who were severely ill, dying/receiving palliative care, and women in labor. As noted by one participant, “Restrictions and procedures relating to patient visits. It’s difficult to not allow patients to see their relatives when they clearly need to spend time with them.” A few participants explicitly stated that it was challenging to defend the hospital’s visitation rules, which they did not agree with, one of whom said, “Visitor restrictions prevented the relatives of seriously ill patients from being close to them. I found it very stressful to have to defend a practice I considered unethical.” Another stated the following:

It’s been difficult to defend nurses’ role during the covid period. It’s difficult to be part of a system that has prevented seriously ill cancer patients from seeing their relatives during hospital stays when they had to undergo major surgical interventions. (Many with uncertain outcomes). I experience moral fatigue. And the sense that those of us who deal with patients are instructed to implement restrictions that have been imposed by people who never have to look patients in the eyes.

#### Digital follow-up

Due to the COVID-19 regulations, HCWs had to provide digital follow-up instead of meeting their patients face-to-face. This was described as ethically challenging by several participants, particularly because they felt that it negatively affected the quality of the assessment and care they could provide.

#### PPE usage

PPE usage led to limited communication, both with patients and their next of kin, and this was described as stressful for the HCWs:

We all have different views regarding how long we’re comfortable visiting a covid patient when wearing infection control equipment (P2–P3 masks). Some would like to visit for a few hours at a time, while others would like to visit for two hours at a time. This leads to stress on my part as I find it difficult to breathe and I develop headaches if I spend longer than two hours indoors. However, if I was to swap with someone who would prefer to stay longer, I tend to push myself to stay longer. This affects both the working day and how I feel when I get home.

#### In-house cohorts

A few participants also described situations related to in-house cohorts. One simply said, “I experienced moral distress in connection with cohort isolation,” whereas others elaborated more and described how such cohorts were challenging not only for the patients (i.e., the rules were very strict in terms of which patients could interact with each other) but also for the staff (i.e., they were not allowed to change cohort even if someone was ill, which was experienced as inflexible and unfair).

#### The general situation

A few participants explicitly stated that they had experienced ECSs that were not specifically related to the pandemic but rather related to the general situation in the hospital:

I experience moral distress often/almost all the time. This has nothing to do with the pandemic. It’s about the number of patients I see and being unable to help patients to the extent they and I expect due to time constraints.

### Unit level

At the unit level, we also identified that the ECSs experienced were related to three main causes: (1) COVID-19-specific issues, (2) treatment level, and (3) mismanagement.

**COVID-19-specific issues** at the unit level included unclear information and routines related to the pandemic, fear of infection, and competence level among reallocated staff.

#### Unclear information and routines

The participants described ECSs related to lack of information and unclear routines during the pandemic, which was time-consuming and frustrating, amongst others because it compromised their ability to care for their patients. For example, one participant said the following:

Colleagues who are afraid of getting sick have tended to interpret precautions and recommendations in the most literal/worst possible sense, even though the infection rates in our region were very low during the first year of the pandemic. I adhered to the rules, but my approach might have been more moderated than normal. I often struggled with how much of the working day was taken up discussing all the various “what if” scenarios and how to interpret the infection control regulations.

Another stated, “There were times when it was difficult to deal with all the infection control procedures at the outpatient clinic and we were forced to come up with our own infection control procedures.” A couple of participants described ECSs related to feeling like a burden on their colleagues due to the COVID-19 regulations (e.g., one nurse was not vaccinated, and her colleagues had to take over all her shifts).

#### Fear of infection

Participants described a fear of becoming infected themselves, of contaminating patients and colleagues, and of bringing infection home to their family. A few leaders described situations in which they could not shield their staff who were elderly, pregnant, or in poor health, from interacting with and treating COVID-19 patients, and asking their staff to come to work even though they expressed concern that they might be infected. As described by one leader:

When employees call and ask whether they should come into work because they are worried they might be sick. They hadn’t experienced any symptoms and the lateral flow test was negative, but they didn’t trust the results. I tell them they have to come in, but I can tell they are scared, nervous, and stressed when they arrive.

#### Reallocated staff’s competence

The participants described situations wherein their reallocated colleagues had not received sufficient training and did not have the necessary competence, as thoroughly described by one nurse:

Major issues with the operation of the intensive care unit between October and January due to the number of covid patients. This meant that general nurses from the ward were re-allocated. These nurses had undergone training in advance and were referred to as helpers. In reality, it is not possible to walk into an intensive care unit to “help.” We carry out advanced treatment and employees need to be trained in order to help. A quick course (without any supervised practice) does not constitute proper training. They were supposed to help me but ended up becoming an additional burden as it became clear they were not suitable for the task. They had replaced adequate help. I was worried about patient safety and found myself on my own with a seriously ill intubated covid patient and a general nurse who was clueless. I wonder if the management (hospital director and clinic management) actually realise what intensive care involves? And will it be my fault if something goes wrong with the patient? It was incredibly stressful and highly provocative.

**Treatment level** Several participants described ECSs related to choice of treatment level, including choosing when to give up intensive care and start palliative care, and considerations related to what is best for the patient versus what is possible to achieve. Many described difficult situations related to what they experienced as overtreatment. For example, one said, “Continuing treatment that should have been discontinued long ago based on my ethical judgement. It’s painful to see patients and relatives suffering. It often leads to the end coming far too late.”

**Mismanagement** included situations related to insufficient qualifications among staff, routines not being followed, and lack of support.

#### Insufficient qualification

Others described situations in which they themselves, or their colleagues, did not have the right qualifications or sufficient training to perform prescribed tasks (without stating specifically that this was related to the pandemic/being reallocated). One of whom related: “Not having the necessary expertise to do a good job for patients.”

#### Routines not followed

A few participants described situations where they felt pressured to violate the ordinary rules and good practice, or noticed that procedures had not been followed, which led to more work and/or unnecessary suffering for patients. For example, one participant stated, “Procedures, such as pressure sore prevention, have not been followed by the departments. This means that unnecessary suffering is inflicted on patients. (…) Turning procedures are not adhered to.”

#### Lack of support

A couple of participants reported lack of support from their leaders and organization, whereof one described situations in which leaders’ decisions hindered HCW’s ability to act ethically. This forced the participant to put themselves on the line, without any guarantee of organizational support:

Also a lack of attention, understanding and support from management when their decisions prevent us from doing what we consider ethically correct in work situations. We end up with our “heads on the block” and with no assurance of support from management, even though it was their decisions that forced us to make ours.

### Patient level

At the patient level, we identified two main causes in the participants’ descriptions of the ECSs experienced: (1) patient autonomy and (2) unvaccinated COVID-19 patients.

#### Patient autonomy

A few participants described ECSs related to overriding their patients’ autonomy, two of whom mentioned testing patients for COVID-19 by force. For example, one participant said, “We have had to test patients using force. We weren’t sure how to manage everything in the beginning. This still doesn’t feel right.”

#### Unvaccinated COVID-19 patients

A few participants described ECSs related to unvaccinated patients. They stated that they found it difficult to stay professional with these patients, that they held a grudge, and experienced resentment towards them. In one participants’ words:

I believe that the unvaccinated intensive care patients were to blame for the country having to go into lockdown. I hold a serious grudge against them, I think they are selfish, and I don’t want to use my expertise on these patients. However, in solidarity with my colleagues, I still did.

## Discussion

The aim of this study was to explore lived experiences of ECS-evoking moral distress during the COVID-19 pandemic, as described by a large sample of HCWs. Among our 977 participants, more than half reported having experienced such situations. This prevalence rate is in line with previous findings^8^ and suggests that ECSs among HCWs was common during the pandemic. We chose to categorize the qualitative descriptions of the ECSs according to the three levels of root causes presented by Hamric and Epstein.^6^ We found that whereas a few participants reported situations caused at the patient level, and some described situations at the unit/team level, the vast majority reported ECSs caused at the system level, predominantly related to resource scarcity, particularly poor staffing.

A common denominator across all three levels of root causes of ECSs described in the present study was that the quality of the patient care had suffered substantially. In particular, the participants reported being overloaded and not having enough time for their patients, including being unable to meet their patients’ most basic needs. They also described difficult decisions regarding who to prioritize for proper monitoring, delayed treatments/long waiting lists, and having to treat patients under suboptimal conditions. In other words, the HCWs described experiences during the pandemic where the prerequisites for delivering adequate medical and psychosocial follow-up of patients and their next of kin were jeopardized, while they remained responsible for ensuring that their patients received best practice care. These findings fit well with the themes covered in the most recent measure developed to explore causes of moral distress among HCWs; the Measure of Moral Distress for Healthcare Professionals (MMD-HP),^7^ particularly the reports of causes at the system level, predominantly related to resource scarcity. That said, there are several themes in the MMD-HP that are not mentioned by our participants, including causes not only related to interactions with the patients’ family (e.g., witnessing HCWs giving “false hope” or accepting family’s insistence to continue aggressive treatment that is not in the best interest the patient) but also related to personal threat to a team member (e.g., fearing retribution for speaking up, power hierarchies, and feeling unsafe/bullied). Albeit speculative, possible explanations for the absence of these themes could be that interactions with family members were limited during the pandemic and/or cultural differences. For obvious reasons, the COVID-19-specific themes we identified in the present study, at all three levels of root causes, are not covered by the MMD-HP.

In line with the study by Gustavsson et al.,^15^ the most prominent cause we identified for ECSs was resource scarcity, particularly poor staffing, but also insufficient bed capacity and lack of equipment. This aligns well with Jameton’s definition of moral distress,^3^ where institutional constraints are described as impeding HCW’s ability to pursue the right course of action, and highlights resource limitation as a major cause of ECSs leading to moral distress. Although these situations were described during the COVID-19 pandemic, it is likely that similar situations occur at other times as well. Notably, a few participants explicitly stated that they had experienced ECSs that were *not* specifically related to the pandemic but rather related to the general situation in the hospital (primarily being overloaded/having too little time). While lack of resources is a well-known challenge in the healthcare sector, it is likely that it became even more pressing during the pandemic, with the lack of buffers in the system coupled with increased workload. Further, some participants described situations at both the unit/team and patient levels which appeared to be more related to day-to-day practice in the hospital than to the pandemic (e.g., mismanagement, difficult choices related to treatment level, and patient autonomy). That said, several participants did explicitly describe situations at all three levels that were unquestionably related specifically to the COVID-19 pandemic and regulations. For example, at the system level, in line with previous literature,^8,12,16^ we found that hospital policies to limit viral transmission (e.g., a restricted family visitation policy) were a source of moral distress. A few participants explicitly stated that it was challenging to defend the hospital’s visitation rules which they found unethical and did not agree with. It is worth mentioning that the primary objective of the pandemic management in Norway was to protect the population from COVID-19 infection and that restrictions and guidelines were developed accordingly.^17^ As noted by Hillestad et al.,^18^ one could argue that minimizing infection was prioritized over all other health-related goals. Consequently, the pandemic regulations largely controlled and restricted the HCW’s work. In line with Hillestad et al.,^18^ our findings vividly illustrate the complexity of the COVID-19 pandemic as a public health emergency, including the challenges that arose when HCWs were forced to balance following regulations (including isolation, PPE usage, and visitation restrictions) with providing what they considered to be proper patient care. In line with previous research,^9,11,12^ PPE usage was described as leading to suboptimal care and inadequate emotional support for patients.

### Strengths, limitations, and future directions

This study contributes significantly to the field of moral distress by systematically describing HCW’s experiences with ECSs during a public health emergency. It is one of a very few studies to use a qualitative approach to explore first-hand accounts of ECS-evoking moral distress among HCWs during the COVID-19 pandemic, and the first to do so with a large, multiprofessional sample. However, certain limitations must be taken into consideration when interpreting the results. First, while the broad recruitment strategy, using various platforms (e.g., e-mails, SMS, online bulletin boards, and posters) likely contributed to a large sample size, it also prevents us from calculating an overall response rate, as we do not know how many potential participants received information about the study. Second, whereas 508 of the participants reported that they had experienced ECS-evoking moral distress during the pandemic, only 323 (64%) provided a qualitative description of these situations, some of which were very brief. Relatedly, few participants described how they had handled these situations. If we had asked the participants about their experiences and coping *face-to-face*, we might have received more, and more elaborate, responses. Third, it may be difficult to clearly distinguish between situations causing purely psychological versus *moral* distress. Hence, even though we presented a definition of moral distress to our participants, is possible that some of the situations described primarily were psychologically, and not necessarily morally, distressing. Fourth, it is possible that the participants’ descriptions of ECSs were biased by the examples provided in the definition of moral distress. That said, only two of the four situations mentioned in the definition were reported by our participants. Finally, the participants were asked a very *broad question*. In a future study, to get more specific information, participants could be asked to describe a single situation in more detail.

## Conclusion

Alleviating moral distress and associated consequences in HCWs is an important aspect of workforce retention.^11^ We found that during the COVID-19 pandemic, a long-term public health emergency, the vast majority of our participants reported ECSs caused at the system level. This is in line with findings from other studies^2,10,11,15^ and strongly indicates that efforts to mitigate moral distress among HCWs should be targeted at the system level. Lack of action at the decision-making level, in terms of enabling HCWs to provide best practice care during crises (e.g., mitigating restriction of visitation for the severely ill, securing sufficient staffing, and staff competence), increases the risk of ECSs among HCWs at all levels. In particular, health authorities and organizations need to build crises preparedness to be able to ensure knowledge-based prioritization for HCWs over time. Further, as pointed out by Klaverkark et al.,^2^ providing a forum for discussing ECSs with colleagues could mitigate moral distress. Also, preparing the HCWs for the difficult tasks they will be asked to carry out, in addition to transparent discussion and acknowledgment when staff/organizational failings have occurred, may be protective and mitigate distress.^19,20^ Of note, compared to the context in the present study, resource limitation was likely more pressing in low-income countries during the pandemic. While we caution the reader to keep cultural context in mind when interpreting the results, we do believe that the causes we identified for ECSs mirror causes relevant for HCWs in other cultural contexts and that our findings may direct further research and serve as a qualified foundation for beneficial interventions across borders.
